# Plant interactions control the carbon distribution of *Dodonaea viscosa* in karst regions

**DOI:** 10.1371/journal.pone.0260337

**Published:** 2021-11-23

**Authors:** Genzhu Wang, Guoyong Tang, Danbo Pang, Yuguo Liu, Long Wan, Jinxing Zhou

**Affiliations:** 1 Jianshui Research Station, School of Soil and Water Conservation, Beijing Forestry University, Beijing, China; 2 Key Laboratory of State Forestry Administration on Soil and Water Conservation, Beijing Forestry University, Beijing, China; 3 Research Institute of Resource Insects, Chinese Academy of Forestry, Kunming, China; 4 Yuanmou Desertification Ecosystem Research Station, State Forestry Administration of China, Kunming, China; 5 Institute of Desertification Studies, Chinese Academy of Forestry, Beijing, China; Ilam University, ISLAMIC REPUBLIC OF IRAN

## Abstract

Biomass and carbon (C) distribution are suggested as strategies of plant responses to resource stress. Understanding the distribution patterns of biomass and C is the key to vegetation restoration in fragile ecosystems, however, there is limited understanding of the intraspecific biomass and C distributions of shrubs resulting from plant interactions in karst areas. In this study, three vegetation restoration types, a *Dodonaea viscosa* monoculture (DM), a *Eucalyptus maideni* and *D*. *viscosa* mixed-species plantation (EDP) and a *Pinus massoniana* and *D*. *viscosa* mixed-species plantation (PDP), were selected to determine the effects of plant interactions on the variations in the C distributions of *D*. *viscosa* among the three vegetation restoration types following 7 years of restoration. The results showed that: (1) plant interactions decreased the leaf biomass fraction. The interaction of *P*. *massoniana* and *D*. *viscosa* decreased the branch biomass fraction and increased the stem and root biomass fraction, but not the interaction of *E*. *maideni* and *D*. *viscosa*. Plant interactions changed the C concentrations of stems and roots rather than those of leaves and branches. (2) Plant interactions affected the soil nutrients and forest characteristics significantly. Meanwhile, the biomass distribution was affected by soil total nitrogen, clumping index and gap fraction; the C concentrations were influenced by the leaf area index and soil total phosphorus. (3) The C storage proportions of all the components correlated significantly with the proportion of biomass. Our results suggested that both the biomass distribution and C concentration of *D*. *viscosa* were affected by plant interactions, however, the biomass fraction not the C concentration determines the C storage fraction characteristics for *D*. *viscosa*.

## Introduction

*Dodonaea viscosa*, a drought tolerant species that has good adaptability to harsh environmental conditions [1], was planted to control rocky desertification in southwestern China starting in the 1980s. Shrubs may be more successful than trees in overcoming drought in karst areas [2]. With the increase in atmospheric carbon (C) dioxide concentrations, forest plantations play an increasingly important role in C sequestration [3]. The biomass and C distributions critically influence forest ecosystem C cycling by shifting the products of photosynthesis between different components [4]. Understanding the biomass and C distribution strategies of plants is crucial to the successful vegetation restoration of degraded lands [5].

Currently, some economic tree species, such as *Eucalyptus maideni* and *Pinus massoniana*, were planted with *D*. *viscosa*, and mixed forests were considered to have higher productivity [6]. However, plant interactions commonly encompass positive and negative effects operating simultaneously and bidirectionally [7, 8]. Mixed plantations can improve the capture, supply or resource use efficiency (e.g. water, nutrients and light) [9]. The processes are often described as facilitative, where the growing conditions are improved; or as competitive reduction, where a less intense interspecific competition replaces intense intraspecific competition [10, 11]. On the other hand, mixed plantations may lead to one species suppressing the growth of another [12]. Furthermore, the majority of variations in plant species traits are associated with the evolutionary adaptation of populations to their enduring local growth conditions [13]. Thus, the biomass and C distribution of the components (leaf, branch, stem and root) of *D*. *viscosa* due to plant interactions requires further understanding.

The plasticity of the plant biomass partition is a key strategy for adapting to various habitats and reflects the evolutionary history of a plant in physical separation resources in the terrestrial environment [14]. The plants have leaves to absorb carbon, roots to absorb soil nutrients and water, and stems and branches to provide mechanical support and to provide a hydraulic pathway [15]; meanwhile, the costs (e.g., biomass partition) and benefits (e.g., light capture) of branches and stems are different [16]. Previous studies have indicated that many factors, such as temperature [17], precipitation [14], altitude [18], vegetation habitat [19], vegetation density [20], and light and soil nutrients [15], have significant effects on plant biomass partition. Poorter et al (2011) suggested that biomass partition to leaves increased with nutrients and decreased with light, whereas the stem fraction increased with densities [15]. Florent et al (2017) indicated that biomass allocation in five semi-arid afforestation species was driven mainly by ontogeny rather than by resource availability [5]. To date, the response of the intraspecific biomass distribution pattern to the environment seems to be different for different species.

Plant C storage is the product of C concentration and biomass. Accurate knowledge of C concentration is essential for converting estimates of forest biomass into forest C stocks [21]. Martin et al (2015) examined variations in wood C concentrations for 17 temperate tree species across five woody tissue types and suggested that intraspecific variations in C across tissue types is less important than interspecific variation [22]. However, investigations by Wang et al (2015) indicated that sites greatly influence the C concentration and storage of white birch in the bole wood, bark, fine roots, and the whole tree scale [13]. Litton et al (2007) suggested C partitioning to foliage, wood and below-ground within a species was sensitive to water and nutrient availability within a site and did not vary with tree density [4]. To date, the results of previous studies on intraspecific C distribution are controversial, and how plant interactions affect biomass and carbon distribution during vegetation restoration in fragile ecosystems remains unclear.

Different vegetation types truly have different effects on soil nutrients [23]. In addition, the shrubs in different vegetation communities are greatly influenced by the competition for resources, such as light, soil nutrients and water. Thus, we hypothesize that the variations in carbon storage fractions of the different components of *D*. *viscosa* are determined both by the variations in biomass fraction and by the variations in carbon concentrations. To test this hypothesis, a *D*. *viscosa* monoculture (DM), a *E*. *maideni* and *D*. *viscosa* mixed-species plantation (EDP), and a *P*. *massoniana* and *D*. *viscosa* mixed-species plantation (PDP) were selected as the investigation subjects. The plantation had been implemented for seven years. Wasteland (W) which had not undergone artificial interference was selected as the comparative subject. The aims of this study were to evaluate: (1) the effects of plant interactions on biomass and on the C distribution of different components (leaf, branch, stem and root) of *D*. *viscosa*; and (2) the effects of the variations in C concentration and biomass fraction on the change in the carbon storage fraction.

## Materials and methods

### Site description

The study site is located in Jianshui County, Yunnan Province, southwestern China (102°33′18″-103°11′31″E, 23°12′42″-24°10′32″N). The annual mean temperature is 18.5°C and the annual mean precipitation is 796.3 mm, the majority of which occurs between April and October. Evaporation rates are almost three times the precipitation rates. Soils in the study area are barren, calcareous and are mainly red and are shallow and discontinuous. Jianshui County belongs to the typical karst graben basin physiognomy, and the karst area is 2,585.16 km^2^.

### Experimental design

In 2009, a 100 ha area of wasteland, which had not been cultivated or planted for more than 50 years, was selected for this study. The native herb vegetation was *Capillipedium assimile*, *Arthraxon hispidus*, *Heteropogon contortus*, *Arundinella anomala*, *Juncus effusus*, *Rubia cordifolia*, *Themeda japonica*, *Imperata cylindrica*, and *Rubia cordifolia*. *D*. *viscosa*, *E*. *maideni* and *P*. *massoniana* were used as the main afforestation tree species. Three treatments with 4,500 trees per ha were arranged, which included two tree and shrub mixed-species plantations and one pure shrub plantation. The DM was planted with 4,500 plants per ha. The PDP was planted with 1,800 *P*. *massoniana* and 2,700 *D*. *viscosa* per ha. The EDP was planted with 2,250 *E*. *maideni* and 2,250 *D*. *viscosa* per ha. Nine plots (20 m × 20 m), which consisted of three replications of DM, PDP and EDP, were selected. All research plots were protected by barbed-wire fences after afforestation; meanwhile, there was no artificial disturbance during the plants’ growing period. The plots were located within 3 km of each other to ensure the same microclimate. After seven years of afforestation, the basic stand characteristics of the research plots are shown in Table 1.

**Table 1:** Characteristics of the sample plots.

Parameter	W	DM	PDP	EDP
Altitude (m)	1,540	1,493	1,480	1,511
Aspect	South	South	South	South
Slope (°)	10	9	11	11
Bare rock rate (%)	52.18	39.67	56.17	44.57
Tree density (Tree/ha)	-	-	1,225	1,656
Shrub density (Tree/ha)	-	3,300	3,120	1,680
Mean tree DBH (cm)	-	-	3.67	6.42
Mean shrub GD (cm)	-	1.36	1.25	2.08
Mean tree height (m)	-	-	5.11	7.06
Mean shrub height (m)	-	1.63	1.37	2.21
Branch height of tree (m)	-	-	0.73	4.11

DBH: diameter at breast height; GD: ground diameter; and Branch height of tree: the height from ground level to the first branch; The numbers refer only to the dominant species of *D*. *viscosa*, *P*. *massoniana* and *E*. *maideni*.

### Investigation and sampling

In July 2016, each plot (20 m × 20 m) was divided into 16 subplots (5 m × 5 m) by white ropes. Four line transects with a length of 20 m through the centre of the subplot in four corners were selected to collect the leaf area index (LAI), clumping index (CI) and gap fraction (GF) through tracing radiation and architecture of canopies (TRAC, 3rd-Wave Engineering, Ottawa, CA) in each plot. All of the TRAC measurements were post-processed using the TRACWin software [24]. Heights and DBH (diameter at 1.3m above ground level) of each tree in the sample plots were measured. *P*. *massoniana* and *E*. *maideni* biomass were calculated using biomass equations established by Chen (2016) [25] and Du (2015) [26] in southern China. Heights, crown area (the maximum width multiplied by the vertical width) and ground diameter (GD, 5cm above ground level) of the total shrubs were surveyed and recorded for each plot. In order to collect the data of biomass and C distribution of *D*. *viscosa*, total 249 *D*. *viscosa* samples in 36 subplots (5 m × 5 m) in the corners, which included four replicates in each DM, PDP and EDP plot, were selected and harvested destructively. The leaf, branch, stem and root biomass of *D*. *viscosa* were measured and recorded in each subplot; the stem height was measured from the ground to the first branch. Component samples of *D*. *viscosa* in each subplot (5 m × 5 m) were collected for the measurement of organic C concentration in the laboratory. Three W plots (20 m × 20 m) were selected to collect the data of soil nutrients and herb and litter biomass. Four 1 m × 1 m subplots were selected in each plot to harvest total herb biomass (above-ground and below-ground) and to collect litter (including all dead plant materials). All vegetation samples were taken to the laboratory and oven-dried at 75°C until a constant.

Four soil profiles near the four corners of the plot were excavated to collect soil samples and to measure bulk density using the cutting ring method for three soil layers. Soil samples were combined into one sample for each soil depth (0–10 cm, 10–20 cm and 20–30 cm layers).

### Laboratory analysis

The organic C concentrations of soil and vegetation samples were obtained using the dichromate oxidation method [27]. Soil total nitrogen (TN) was measured using an element analyzer (Vario EL III, Element, Germany), and total phosphorus (TP) was obtained using fused sodium hydroxide with the molybdenum stibium anti-regent color method [28]. Soil pH, natural water content and bulk density were measured at the Institute of Soil Science, Chinese Academy of Sciences [23].

### Statistical analysis

Date for 9 groups (3 treatments × 3 replicates) per case of forest characteristics, soil nutrients, biomass and C distribution traits of *D*. *viscosa* were tested for normal distribution and variance homogeneity and log-transformed to satisfy the normality of distribution and homogeneity of variance. One-way ANOVA and least square difference (LSD) multiple comparison tests were used to assess the differences in forest characteristics, biomass and C allocation traits at *P*<0.05. Multiple linear regressions were applied to explore the relationships among C storage fraction, biomass fraction and C concentration. The one- way ANOVA tests and multiple linear regressions were performed using SPSS 18.0 (SPSS Inc., Chicago, IL, USA). The graphs were plotted using Origin 9.0.

We applied a redundancy analysis (RDA) to explore the association between environmental factors and variations in C concentration and biomass fraction of different components (e.g. leaf, branch, stem and root). Canoco 5.0 (Centre for Biometry, Wageningen, The Netherlands) was used to carry out RDA and test the proportion of variation attributable to environment effects, interaction effects, and unexplained effects [29]. The Monte Carlo permutation method, based on 999 runs with randomized data was used to determine the statistical significance of RDA. If P <0.05, the results were considered to be statistically significant.

## Results

### Plant and soil nutrient characteristics of sample plots

Compared with W, the soil C concentration in DM significantly increased, while no difference was found among W, PDP and EDP (Table 2). The soil TN and TP decreased significantly in DM, PDP and EDP compared with W and were lowest in PDP among the three plantation types. The TN was higher in DM than that in EDP, however, the TP was lower in DM than that in EDP. The soil water content was higher in PDP; there was no difference among W, DM and EDP. The *E*. *maideni* was significantly higher than *P*. *massoniana*, meanwhile the first branch height from the ground level for *E*. *maideni* was significantly higher than for *P*. *massoniana* (Table 1). The total vegetation biomass in the three plantation restoration types all increased greatly compared with the W, while the total biomass of EDP and PDP recorded the highest level and were greatly higher than that of DM. The LAI of EDP was highest among the three treatments, while it was higher for DM than for PDP. The CI of DM was highest, while the CI was higher for PDP than for EDP. The GF of PDP was significantly lower than for DM and for EDP, while there was no difference between DM and EDP.

**Table 2:** Environmental factors of the sample plots after 7 years afforestation.

Parameter	W	DM	PDP	EDP
LAI	-	0.91±0.08b	0.61±0.07c	1.91±0.13a
CI	-	0.85±0.07a	0.75±0.06b	0.67±0.04c
GF (%)	-	30.2±2.8a	21.4±1.9b	29.6±3.1a
pH	5.91±0.08b	6.21±0.16a	6.02±0.10ab	5.58±0.30c
SWC (%)	32.86±0.81b	34.14±2.02b	47.86±2.28a	31.02±1.42b
BD (g/cm^3^)	1.11±0.02b	1.18±0.02a	1.14±0.01b	1.14±0.02b
SOC (g/kg)	34.83±15.36b	58.01±3.23a	32.10±1.81b	41.47±8.74ab
TN (g/kg)	3.31±0.09a	1.48±0.01b	1.09±0.01d	1.23±0.09c
TP (g/kg)	1.73±0.14a	0.68±0.01c	0.53±0.01c	1.03±0.12b

Means and standard deviations of leaf area index (LAI), clumping index (CI), gap fraction (GF), pH, soil water concentration (SWC), bulk density (BD), soil organic carbon concentration (SOC), total nitrogen (TN) and total phosphorus (TP). Different lowercase letters denote a significant difference among the different vegetation types at *p* < 0.05.

### Biomass and C distribution of *D*. *viscosa* in different plantation types

The average biomass of a single plant of *D*. *viscosa* in EDP was much higher than that in DM and PDP (Table 3). The leaf biomass fraction (LBF) in DM (20.87%) was significantly higher than that in PDP (14.57%) and in EDP (16.93%), while no difference was found between PDP and EDP. The branch biomass fraction (BBF) in PDP (18.98%) was lower than that in DM (27.89%) and in EDP (30.01%), while no difference was found between DM and EDP. The stem biomass fraction (SBF) and root biomass fraction (RBF) in PDP (40.32% and 26.13%) were all significantly higher than those in DM (31.19% and 20.05%) and EDP (31.64% and 21.42%), whilst there was no difference between DM and EDP.

**Table 3:** Biomass and carbon storage of single plants of *D*. *viscosa* for different components in different vegetation types.

Components	DM (g)		PDP (g)		EDP (g)	
	Biomass	Carbon storage	Biomass	Carbon storage	Biomass	Carbon storage
Leaf	316.5±30.2a	192.2±18.23a	221.8±17.26b	134.6±11.55b	315.4±28.77a	184.5±15.69a
Branch	423.0±35.3b	240.2±21.01b	289.0±24.32c	163.5±16.21c	559.1±49.23a	351.2±28.79a
Stem	473.0±39.4b	261.26±22.4b	613.8±59.8a	339.7±32.7a	589.5±52.3a	392.9±37.6a
Root	304.1±18.7b	150.2±14.6b	397.8±37.8a	205.1±19.4a	399.1±17.6a	238.1±19.8a
Total	1516.5±130.2b	867.9±85.4b	1522.4±139.7b	842.9±79.6b	1863.1±165.8a	1166.7±103.2a

Means and standard deviations. Different lowercase letters denote significant differences among different vegetation types at *p* < 0.05.

**Fig 1:**
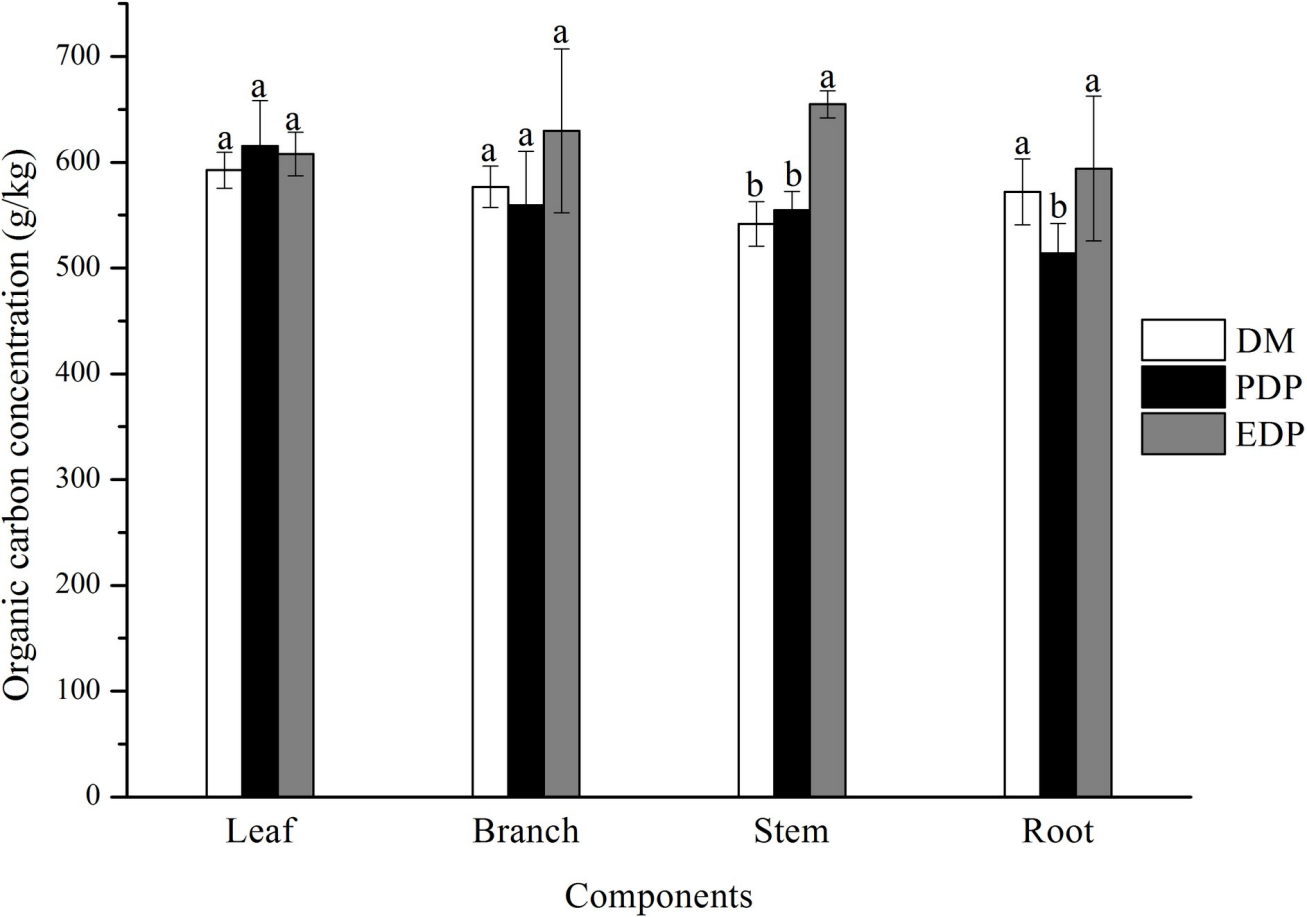
Carbon concentrations (g/kg) of different *D*. *viscosa* components for different vegetation types. Different lowercase letters indicate significant differences among the different vegetation types at *p* < 0.05. Vertical bars represent standard deviations.

The leaf C concentration (LCC) and branch C concentration (BCC) showed no difference among the three treatments (Fig 1). The stem C concentration (SCC) in EDP (654.89 g/kg) was significantly higher than those in PDP (554.66 g/kg) and DM (541.72 g/kg), and no difference was found between PDP and DM. Root C concentrations (RCC) in PDP (513.9 g/kg) were significantly lower than those in DM (572.16 g/kg) and EDP (594.1 g/kg), while there was no difference between DM and EDP.

The C storage levels of single plants of *D*. *viscosa* in DM (867.9 g) and PDP (842.9 g) were significantly lower than those in EDP (1,166.7 g). The leaf C storage proportion in DM (22.05%) was significantly higher than those in PDP (16.25%) and EDP (16.4%). The C storage proportions of stems and roots in PDP were all significantly higher than those for DM and EDP, whilst branch C storage proportions in PDP (19.17%) were significantly lower than those in DM (27.71%) and for EDP (30.2%). No differences in branches, stems and roots C storage proportions were found between DM and EDP.

### Relationships between environmental factors and biomass and C distribution

**Fig 2:**
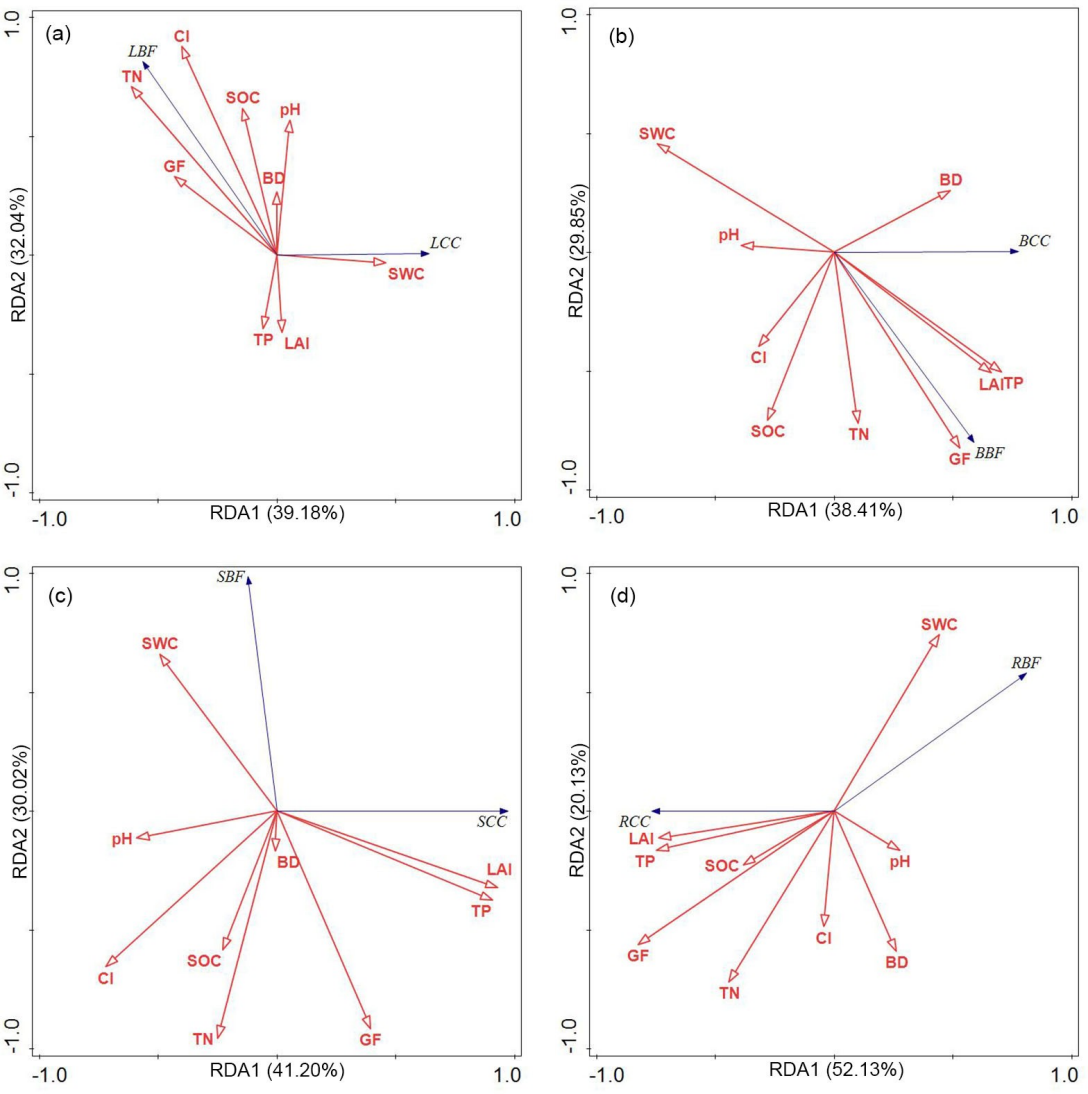
Ordination diagrams generated by redundancy analysis of the effects of environmental factors on biomass fraction and carbon concentration of leaf (a), branch (b), stem (c), root (d). Purple arrows represent biomass fraction and carbon concentration; red arrows represent environmental factors. CI: clumping index; LAI: leaf area index; GF: gap fraction; SOCD: soil organic carbon concentration; TP: total phosphorus; TN: total nitrogen; SWC: soil water content; BD: bulk density; LBF: leaf biomass fraction; LCC: leaf carbon concentration; BBF: branch biomass fraction; BCC: biomass carbon concentration; SBF: stem biomass fraction; SCC: stem carbon concentration; RBF: root biomass fraction; RCC: root carbon concentration.

Only CI and TN showed significant positive correlations with LBF (Fig 2A). The CI and TN can explain 69.6% of the variation (S2 Table). The BBF was only significantly negatively correlated with the GF (Fig 2B), which can explain 69.3% of the variation. The SBF and RBF both decreased significantly with increases in GP and TN (Fig 2C and 2D). The variation of the SBF can be explained 68.3% and the RBF can be explained 64.1%.

The LCC and BCC were not significantly influenced by environmental factors, while the SCC and RCC increased significantly with increases in LAI and TP. The variation in the SCC can be explained 52.1% and the RCC can be explained 65.5%.

The C storage fraction was significantly correlated with biomass fraction for all components, and there was no significant correlation between the C storage fraction and the C concentration for all components, which was not consistent with the hypothesis (Table 4).

**Table 4:** Multiple linear regressions of carbon storage fraction of different components.

Components	n	Equations	R^2^	F	P
Leaf	9	y = 0.916x1+0.067x2	0.942	95.014	0.000
Branch	9	y = 0.994x1+0.014x2	0.998	959.312	0.000
Stem	9	y = 0.938x1+0.245x2	0.995	2469.789	0.000
Root	9	y = 0.958x1+0.126x2	0.955	274.315	0.000

y: carbon storage fraction, x1: biomass fraction, and x2: carbon concentration.

## Discussion

With the vegetation restoration, the soil nutrients were substantially extracted to forest productivity [30, 31]. The input of soil nutrients mainly derived from the decomposition of the litter and fine roots of vegetation in plantations [32, 33]. Soil nutrients (ie. SOC, TN and TP) maybe decreased in the initial afforestation stage resulting from the higher consumption of soil nutrients by plants than the input of soil nutrients [34], which was same with our results, that the selected soil nutrients in three plantation restoration types were lower than those in W. Three possible reasons may explain outcomes of this study resulting from plant interactions. First, litter rather than root interaction accelerated nutrient cycling in certain regions with arid climate and rocky soil [35]. Litter fall from evergreen broad-leaved tree species (*E*. *maideni*) were greater than that from *P*. *massoniana* (S1 Table). Second, *E*. *maideni* and *D*. *viscosa* are angiosperms, while *P*. *massoniana* is a gymnosperm. Litter from gymnosperms contain high contents of lignin, cellulose and secondary metabolites, leading to the slower decomposition rate of litter from gymnosperms than that from angiosperms [36], which accounted for the lower soil nutrients concentration in PDP compared to the other two plantations. Moreover, the productivity of DM was significantly lower than that of PDP and EDP, resulting in the lower consumption of soil nutrients in DM. Our results indicated LAI of broad-leaved forests was higher than that of coniferous forests, and broadleaf mixed plantations had higher LAI than pure shrub forests.

Vegetation biomass is affected by environmental factors such as water, heat and soil conditions [26, 37]. The distribution patterns of vegetation biomass have plastic responses to their environmental conditions, relating to resource stress [38]. The plasticity of the LBF was primarily influenced by light and soil nutrients [15]. Poorter et al (2011) indicated that the LBF increased strongly with nutrients, which can explain the outcome that LBF of *D*. *viscosa* increased mainly with the TN. Meanwhile, plants grown in lower light conditions may show a higher LBF to maximize the light resource for the same growth period, which differed from our results [15]. In the contrast, low light levels under the tree crowns with stand growth can result in the shedding of shade-intolerant leaves at the base of the canopy [17], which may explain our results that the LBF of *D*. *viscosa* increased with the CI. We therefore deduce that some leaves of *D*. *viscosa* may fall off due to the effects of crowns of *P*. *massoniana* or *E*. *maideni*.

Branch traits were generally affected by spacing, and the branch biomass usually decreased with the decreased tree spacing, which was beneficial to the stem [39]. The growth and production of branches were likely stimulated by light availability [40], which was the same as our results that the BBF of *D*. *viscosa*, which increased with the GF. This finding may also explain why the stem biomass storage proportions were negatively correlated with the GF. This result therefore concurs with others [39], but was contrary to the results reported by Zhang et al. (2012) showing increasing stem ratios with self-thinning [20]. These differences may be related to growth stage of the forest. In mature forests, trees tend to towards a radial growth pattern to obtain light resources for the relatively high vegetation coverage, resulting in a higher proportion of stems [20].

Plants may allocate more biomass to roots to maximize uptake of nutrients and water resources in conditions with shortages of soil nutrients and in arid environments [38]. According to the optimal partitioning theory [41], the result, that the RBF negatively correlated with TP and TN rather than with SWC indicated that the growth of *D*. *viscosa* was limited by soil nutrients rather than by water availability. Mattia et al (2016) found that canopy cover reduction increased the fine roots in beech, which was similar with our result that the GF positively correlated with the RBF [42]. Increased solar radiation reaching the ground surface and the consequent increase in soil temperature might have influenced the cambial activity and successive xylogenesis [42].

The variations of C concentrations for a given tree species in different communities reflect the change in its chemical makeup [43]. Mattia et al (2016) found that forest canopy reduction stimulates xylem production and lowers carbon concentrations in the fine roots [42]. This observation was similar to our findings that stem and root carbon concentrations decreased with decreasing LAI. The reason may be that the ratio of cellulose to lignin concentrations increased with increases in the biomass of stems [44] and roots [45]. Furthermore, the C concentration of the stems and roots all increased with the TP. The outcomes may also result from the lower level TP causing an increase in the fraction of stems (even not significantly) and roots by increasing the secondary xylem, which had lower C concentration [42]. Therefore, our results, along with those of others, support the idea that stem and root carbon concentrations are positive related to the growth traits [21, 46].

Litton et al (2007) suggested that biomass partition should not imply C storage fractions of aboveground and belowground components [4], which was different from our results, which showed that C storage fractions significantly correlated with biomass fractions of all components of the *D*. *viscosa*. The reason may be that the research subject selected by Litton (2007) was consisted by different trees from different ecosystems under various resource availabilities, stand ages and levels of competition [4]. These results indicated that the variations in intraspecific C concentrations did not significantly influence the C fractions of different components, which is similar to Martin et al (2015), who suggested that intraspecific variations in C across tissue types are less important than interspecific variation [22].

## Conclusions

In general, plant interactions controlled the environmental factors for *D*. *viscosa* and then substantially changed the biomass and carbon distribution. The biomass fractions of all the components and the C concentrations of the stems and roots of *D*. *viscosa* were changed by plant interactions. However, the C storage distribution characteristics were determined by biomass rather than by the C concentration. The *D*. *viscosa* could change its biomass and carbon distribution patterns to adapt the interaction with other plants.

## Supporting information

S1 Table Vegetation biomass (t/hm2) in the different vegetation restoration types. (DOCX)

S2 Table Individual contribution of primary parameters to the proportion (%) of variation explained in biomass and carbon concentration of different components. (DOCX)

S1 Data (XLSX)
