# Supplementary material for: Plant interactions control the carbon distribution of Dodonaea viscosa in karst regions
Source: PLoS One. 2021 Nov 23;16(11):e0260337. doi: 10.1371/journal.pone.0260337 (PMC8610255; doi:10.1371/journal.pone.0260337)
Supplement: S1 Table — (DOCX) [file pone.0260337.s001.docx]

**Table S1.** Vegetation biomass (t/hm^2^) in the different vegetation restoration types.

| **Components** | **W** | **DM** | **PDP** | **EDP** |
| --- | --- | --- | --- | --- |
| Tree layer |  |  |  |  |
| Leaf | - | - | 0.27±0.03b | 1.88±0.27a |
| Branch | - | - | 5.04±0.21a | 2.96±0.13b |
| Stem | - | - | 11.08±1.13a | 14.24±1.53a |
| Root | - | - | 1.63±0.06b | 1.32±0.21a |
| TB | - | - | 18.01±0.58a | 20.4±3.75a |
| Shrub layer |  |  |  |  |
| Leaf | - | 1.05±0.18a | 0.69±0.05b | 0.53±0.04b |
| Branch | - | 1.41±0.15a | 0.90±0.07a | 0.94±0.08a |
| Stem | - | 1.58±0.16a | 1.92±0.18a | 0.99±0.09b |
| Root | - | 1.01±0.11a | 1.24±0.12a | 0.67±0.07b |
| SB | - | 5.05±0.46a | 4.75±0.43a | 3.13±0.31b |
| Herb layer |  |  |  |  |
| Aboveground | 1.50±0.33a | 1.66±0.98a | 2.54±0.87a | 1.38±0.68a |
| Belowground | 0.88±0.15a | 0.86±0.44a | 0.97±0.21a | 0.73±0.21a |
| Subtotal | 2.38±0.53a | 2.52±2.26a | 3.51±1.09a | 2.11±0.92a |
| Litter layer | 0.62±0.14d | 1.92±0.42c | 3.82±1.02b | 6.01±1.23a |
| Total Vegetation |  |  |  |  |
| Aboveground | 2.12±0.45c | 7.62±1.53b | 26.25±3.63a | 28.93±2.25a |
| Belowground | 0.88±0.30c | 1.87±0.75b | 3.84±0.45a | 2.72±0.51a |
| TVB | 3.00±0.81c | 9.49±2.45b | 30.09±4.46a | 31.65±2.89a |

Means and standard deviations. TB: tree layer biomass, SB: shrub layer biomass, and TVB: total vegetation biomass. Different lowercase letters denote significant differences among different vegetation types at *P*<0.05.
