# Supplementary material for: Plant interactions control the carbon distribution of Dodonaea viscosa in karst regions
Source: PLoS One. 2021 Nov 23;16(11):e0260337. doi: 10.1371/journal.pone.0260337 (PMC8610255; doi:10.1371/journal.pone.0260337)
Supplement: S2 Table — (DOCX) [file pone.0260337.s002.docx]

**Table S2** Individual contribution of primary parameters to the proportion (%) of variation explained in biomass and carbon concentration of different components.

| **Parameter** | **LBF** | **LCC** | **BBF** | **BCC** | **SBF** | **SCC** | **RBF** | **RCC** |
| --- | --- | --- | --- | --- | --- | --- | --- | --- |
| LAI | - | - | - | - | - | 24.2 | - | 33.2 |
| CI | 27.5 | - | - | - | - | - | - | - |
| GF | - | - | 69.3 | - | 30.2 | - | 22.3 | - |
| TN | 20.4 | - | - | - | 32.1 | - | 13.1 | - |
| TP | - | - | - | - | - | 18.3 | 8.7 | 21.3 |
| Interactions | 21.7 | - | - | - | 5.6 | 9.6 | 20 | 10.6 |
| Unexplained | 30.4 | 100 | 30.7 | 100 | 31.7 | 47.9 | 35.9 | 34.5 |

LBF: leaf biomass fraction; LCC: leaf carbon concentration; BBF: branch biomass fraction; BCC: biomass carbon concentration; SBF: stem biomass fraction; SCC: stem carbon concentration; RBF: root biomass fraction; RCC: root carbon concentration.
